# Paleo-geomorphic features of pre-Jurassic and its oil-controlling effect in Wuqi–Dingbian area

**DOI:** 10.1038/s41598-024-60510-y

**Published:** 2024-05-08

**Authors:** Guicheng Wang, Xiaolin Wang, Licheng Xie, Ruili Huang

**Affiliations:** 1 Xi’an Shiyou University, Xi’an, China; 2 China University of Petroleum (Beijing), Beijing, China; 3 Exploration and Development Research Center of Yanchang Oilfield Co., LTD, Xi’an, China

**Keywords:** Pre-Jurassic, Paleogeomorphic features, Oil-controlling function, Accumulation model, Fossil fuels, Energy science and technology

## Abstract

The Fuxian-Yan10 layers are the main oil-producing reservoirs of Jurassic in Wuqi–Dingbian area of Ordos Basin. However, due to the lack of understanding of the pattern and distribution characteristics of oil reservoirs, the benefits of exploration and development are restricted. In order to provide theoretical guidance for the study of similar geological features, based on the analysis of paleogeomorphic features and evolution, the analysis focuses on the influence of palaeo-geomorphology on oil reservoir distribution, and summarizes the main types of reservoir models in the study area. The results show that there are four types of palaeo-geomorphic units in the Wuding area: palaeo-river, slope, highland and interriver hill. In the study area, the Jurassic paleogeomorphology controls the sedimentary development and distribution from Fuxian Formation to Yan 9 Formation. The compacted structure and lithologic barrier provide good trapping conditions for the paleogeomorphic oil. Moreover, the swampy coal measures and mudstone at the top of Yan 9 play a sealing role for oil accumulation, and the bottom water was obviously driven. In addition, the pre-Jurassic deep valley was the main channels for oil migration. On this basis, it is concluded that there are four reservoir-forming models in Wuding area: slope type, river hill type, ancient river type and highland type.

## Introduction

The Ordos Basin is an important energy producing area in China. The exploration of oil and gas in the basin has a wide range and many exploration horizons from the Lower Paleozoic to the Middle Cenozoic, and the exploration objects are extended from anticlinal to subtle oil and gas reservoirs^1,2^. In recent years, with the deepening of exploration and development, major breakthroughs have been made in the exploration of Jurassic in the inner and marginal areas of the basin. The Jurassic reservoir is a large valley filling type eroded palaeoriver reservoir, which has the characteristics of low development cost and good comprehensive benefits^3^. Such reservoirs are controlled by various factors, but the lower Jurassic reservoirs are mainly controlled by pre-Jurassic palaeogeomorphology. At the end of the Indosinian movement, the entire basin has been uplifted and denuded, forming a number of dendritic ancient rivers. In the early Yanshanian Movement (Early Middle Jurassic), the study area was in a weak extensional tectonic background, with regional subsidence and wide sedimentary range, and mainly developed river and delta sedimentary systems.

With the continuous exploration and development of Jurassic in Wuding area, new progress has been made in petroleum exploration of Jurassic in this area. Exploration research shows that the pre-Jurassic palaeogeomorphology is an important geological basis for controlling the reservoir distribution in Yan'an Formation^4,5^. The distribution, accumulation model, and trap conditions of high-quality Jurassic reservoirs in the study area are complex. Previous studies on the Jurassic have mainly focused on paleogeomorphic features, hydrocarbon generation, storage and other single factor analysis^6,7^. However, the relationship between paleogeomorphic features and reservoir-forming rules and patterns was not adequately explored, making trap prediction difficult and severely restricting oil and gas exploration and development. Based on the basic analysis of paleogeomorphology evolution, this study reveals the control effect of paleogeomorphology on reservoir types and distribution mechanism. The aim is to provide a theoretical basis for the study of similar geological characteristics.

## Regional geological overview

**Figure 1:**
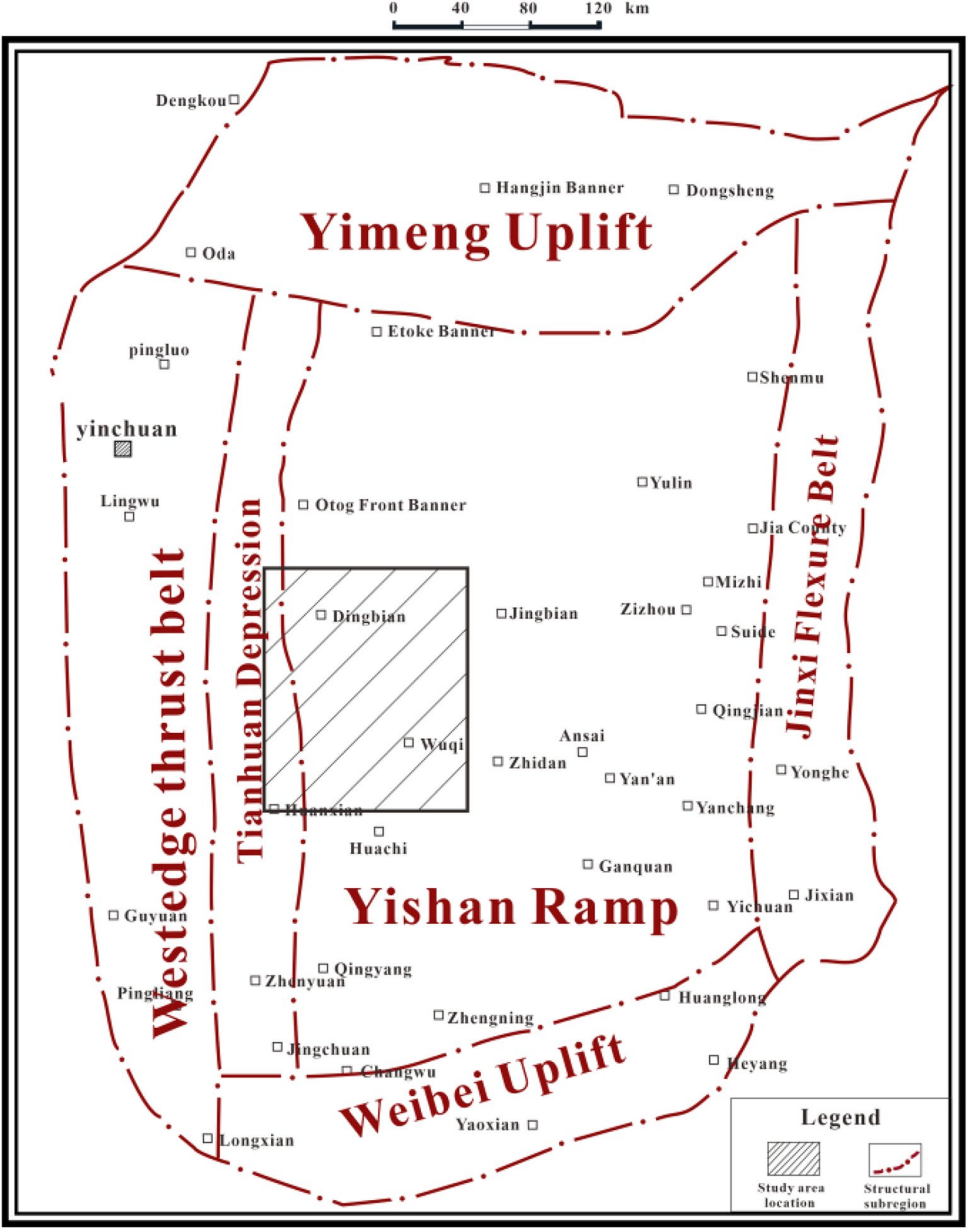
Location map of the study area (Coreldraw, 18.0, https://www.coreldrawchina.com/).

Research area is located in Dingbian and Wuqi counties of Shaanxi Province. It is structurally located in the middle and western part of the Yishaan slope of the Ordos Basin (Fig. 1), with an area of about 1.2 × 10^4^ km^2^. The surface of the research area is a typical loess source such as landforms, with undulating topography and gullies in the regional surface, and the ground elevation is mostly in the range of 1220–1900 m, with a large relative height difference.

The main Jurassic oil-bearing formation system in the study area is the Yan'an Formation. The Yan 10 section in this area is a typical braided river deposit with a large thickness of sand body, and its lateral connectivity is enhanced compared with the Fuxian Formation. Due to the undercutting of the river, the thickness of the Yan 10 formation also varies in size. The Yan 9 section is mainly developed with lakes, delta plains and delta front deposits, and the thickness of the sand body is thin at 10–20 m. The thickness of the strata is more stable at 30–45 m. Through the study of light and heavy mineral fractions in the study area, the main source comes from the northwest direction, and the sand body spreads along the north-west-south-east direction. The sand body spreads along the north-west-south-east direction.

## Paleogeomorphic restoration and its features

### Methods of paleogeomorphic restoration

There have been many studies on the pre-Jurassic paleomorphology of the basin, and some understanding has been achieved in the reservoir formation mechanism and reservoir distribution law. However, with the continuous addition of new data and the development of new technology in recent years, the research results of the previous research can hardly meet the needs of present-day scientific research and production, so it is necessary to finely delineate the pre-Jurassic paleomorphology of the Wuding area to provide a more accurate geological basis for the next reservoir prediction of the Jurassic system.

This study on the restoration of the pre-Jurassic paleomorphology in Ordos Basin integrates the paleogeological map method, geophysical method, impression method, sand thickness method and other geological analysis methods, and the multi-methods are integrated and deepened step by step to finely portray the morphological characteristics and paleomorphological evolution of the pre-Jurassic paleomorphology.

#### Paleogeological map method

**Figure 2:**
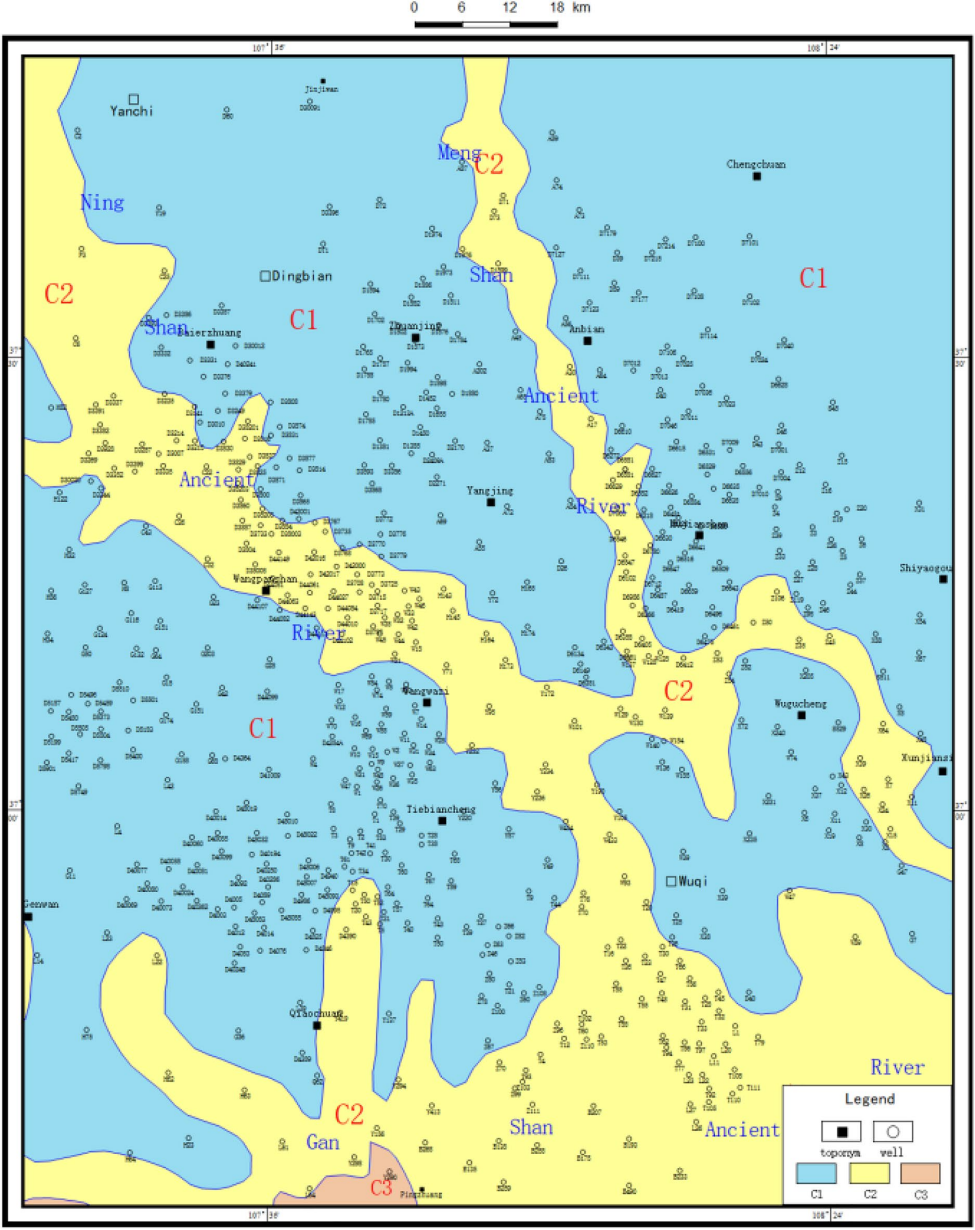
Pre-Jurassic paleogeological map of Wuqi–Dingbian area (Coreldraw, 18.0, https://www.coreldrawchina.com/).

The paleogeological map method is mainly used to understand the regional tectonic pattern before deposition. The specific steps are to understand the regional paleotectonic pattern and stratigraphic outcrop before deposition by using the paleogeological map and stratigraphic residual thickness map, so as to understand the stratigraphic plan and spatial spreading characteristics as a whole, and to characterize the paleomorphic features according to the denudation of strata [8]. Based on the results, it can be seen that the study area is missing the Chang 2 stratum in the palaeochannel development area and the Chang 1 stratum in the rest of the area (Fig. 2).

#### Impression method

The impression method is to spread the straum thickness on the plane, outline the thickness contour map, select the classification criteria of each geomorphic unit, and subdivide the geomorphic unit, which can better reflect the morphology and spread of ancient rivers in the ancient river deposition period^9^. The sediments of Yan'an Formation are mainly channel sand bodies in the downcut valley, with low compaction rate. The top of Yan 9 can be regarded as a base plane when Yan 9 began to fill up, and the "impression method" is applied for paleomorphological restoration.

The stratigraphic thickness between the erosion surface of the top of the Yanchang Formation and the top coal of Yan 9 Formation indirectly reflects the undulating pattern of the erosion surface. The paleotopography is high, so the overlying sediment is thin; The paleotopography is low, so the overlying sediment is thick. In addition, the thickness of Yan 9 to Fuxian is a mirror image of the pre-Jurassic paleomorphology. The thickness can be used to invert the pre-Jurassic paleomorphology pattern, and the thickness of strata from large to small reflects the paleomorphology from low to high.

**Figure 3:**
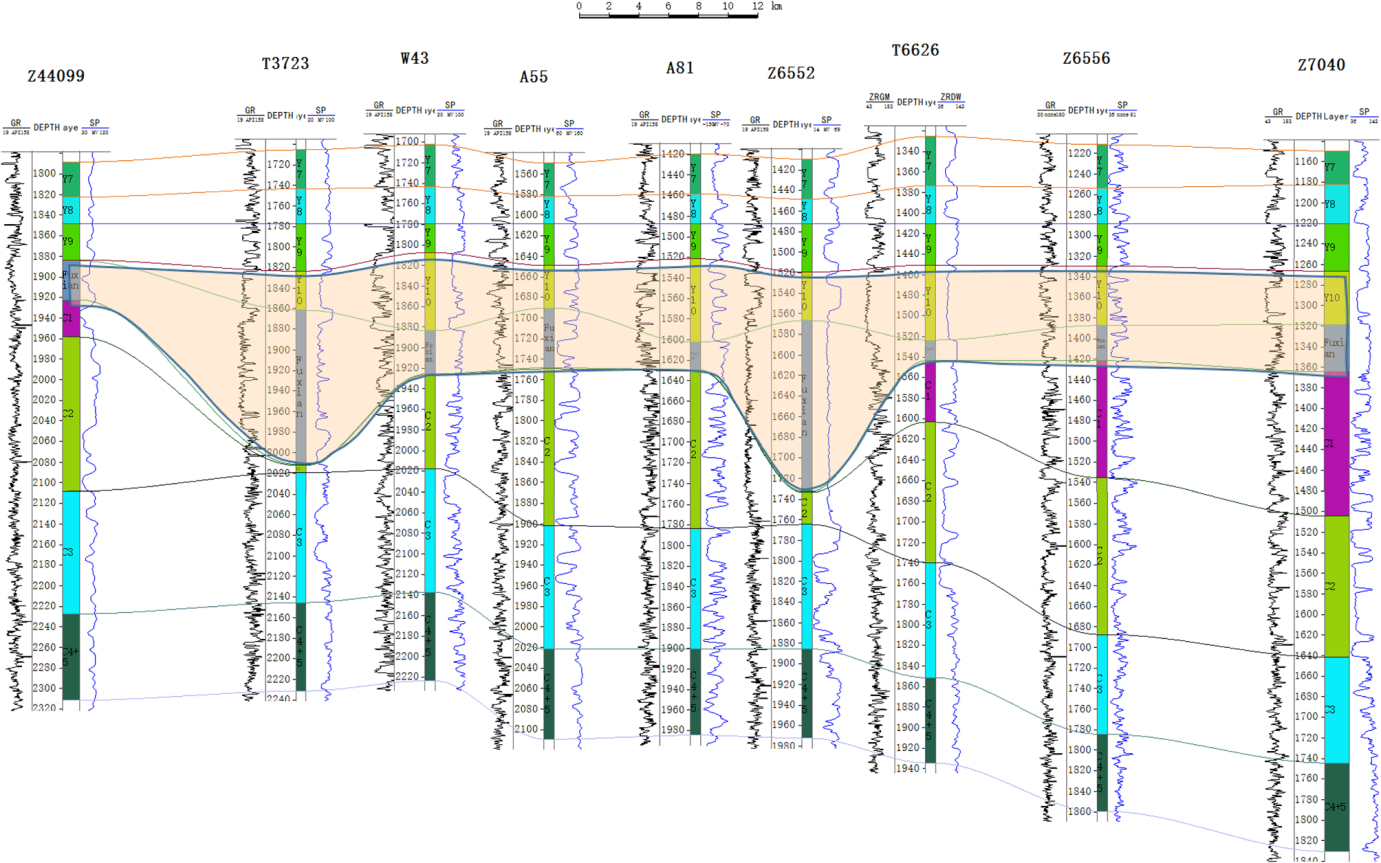
Comparison of the stratigraphy of well 44099—well Zizi 7040 from Chang 4 + 5 to Yan 7 (Yan 9 top pulling flat).

After the flattening of the top of Yan 9, its lower paleotopography can be reflected according to the thickness variation of the Yan10 + Fuxian strata: the places with large thickness reflect the lower part of its lower paleotopography, that is, the location of the paleosol; while the places with small thickness coincide with the higher part of the paleotopography. The thickness of the single positive rotational stratum in the river channel is also indicative of the paleowater depth to some extent (Fig. 3).

#### Sand body thickness method

**Figure 4:**
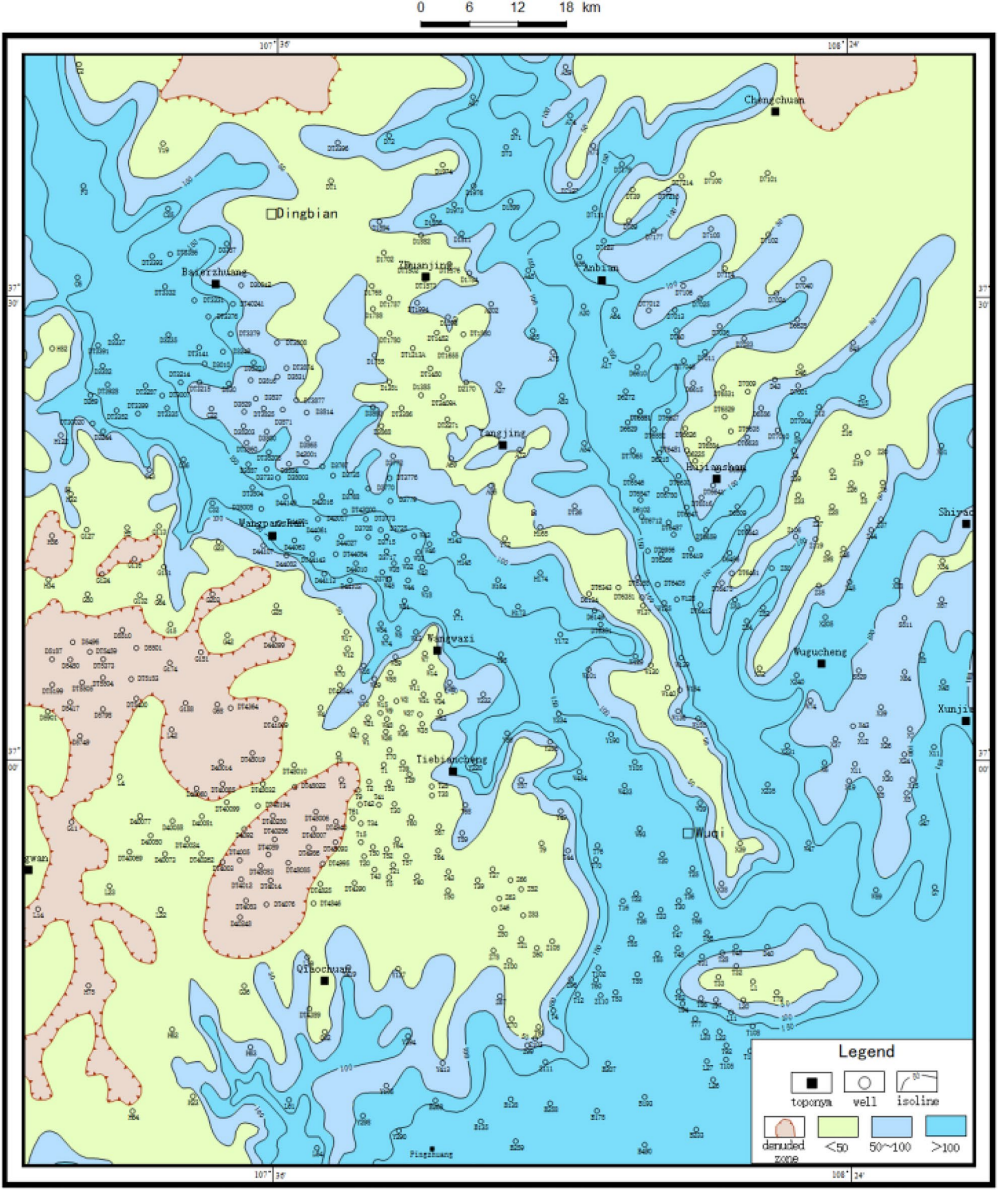
Contour map of stratigraphic thickness of Yan 10 + Fuxian in Wuqi–Dingbian area (Coreldraw, 18.0, https://www.coreldrawchina.com/).

**Figure 5:**
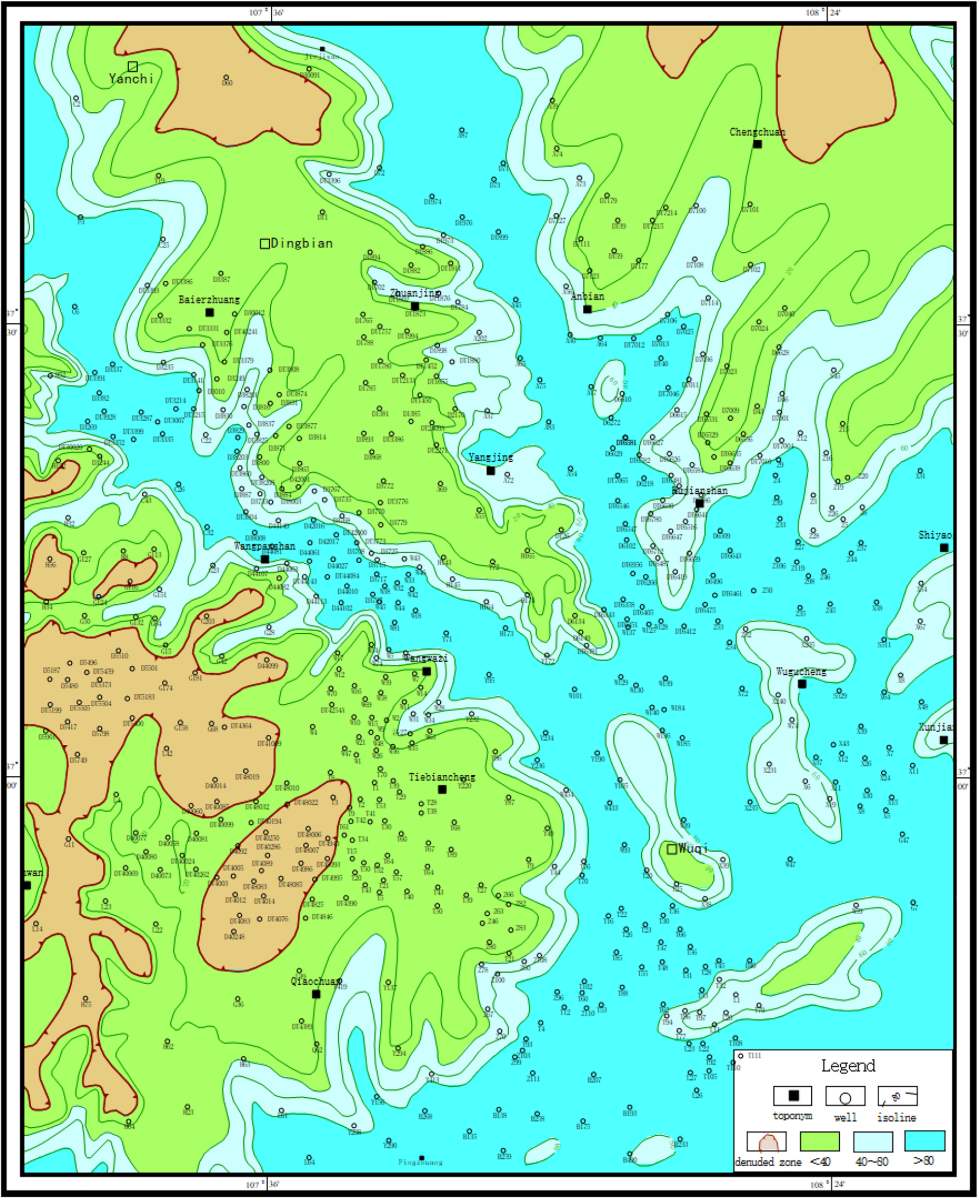
Contour map of sandstone thickness in Yan 10 + Fuxian in Wuqi–Dingbian area (Coreldraw, 18.0, https://www.coreldrawchina.com/).

The cumulative thickness map of the stratigraphic sandstone can outline the denuded and deposited areas, show the development degree and background conditions of the alluvial system, and help to recover the petrographic paleogeographic map^10^. In the petrographic paleogeographic map, the development type characteristics and hydrodynamic conditions of the sedimentary system at that time can be discerned. At the same time, the spatial and temporal arrangement of the early Jurassic strata can be seen from the lithofacies paleogeographic maps. In addition, the variation trend of terrain can also be indicated according to the variation trend of medium thickness line of sandstone isopath map and stratum isopath map. It is found by statistics that the area with strata thickness of more than 100 m in Yan10 + Fuxian county generally represents the ancient water system area. The thickness of the sedimentary sand body can accurately reflect different paleogeomorphic units and their distribution (Figs. 4 and 5). For example, the thickest place on the isothickness map of Yan10 + Fuxian sandstone is often the location of the ancient valley.

#### Three-dimensional morphological portrayal of paleolandscapes

**Figure 6:**
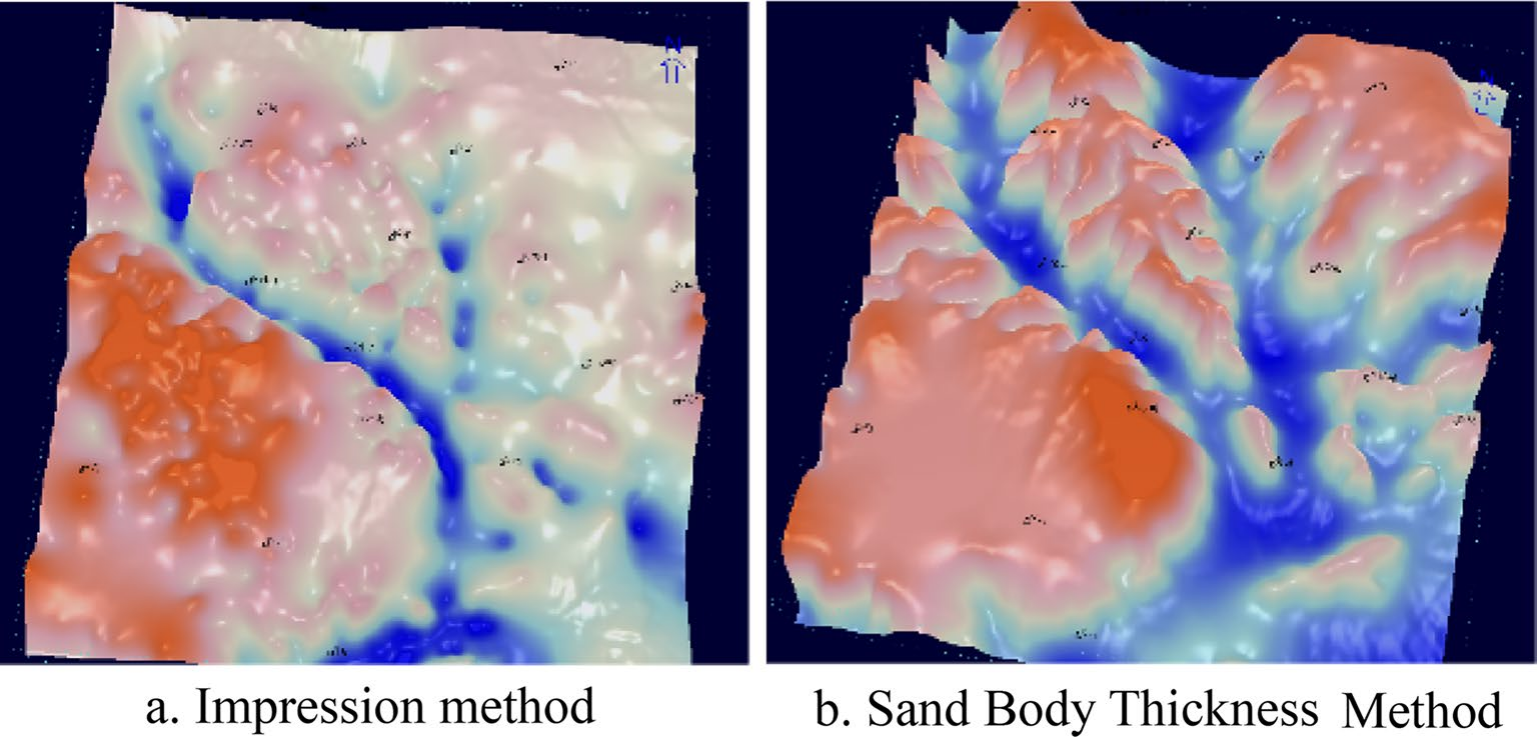
Ancient landform engraving of Yan 9 ~ Fu County in Wuqi–Dingbian area.

Due to the influence of tectonics, material supply and other factors, the quasi-plainification at the end of Yan 9 deposition is uneven, resulting in the paleomorphic morphology carved by the impression method is not fine enough in some palaeochannels and slope areas. However, the paleomorphic gullies carved by the sand thickness method are crisscrossed. The boundaries and distribution of paleomorphic units such as uplands, slopes and inter-river mounds are clear (Fig. 6).

### Paleolandscape features

**Figure 7:**
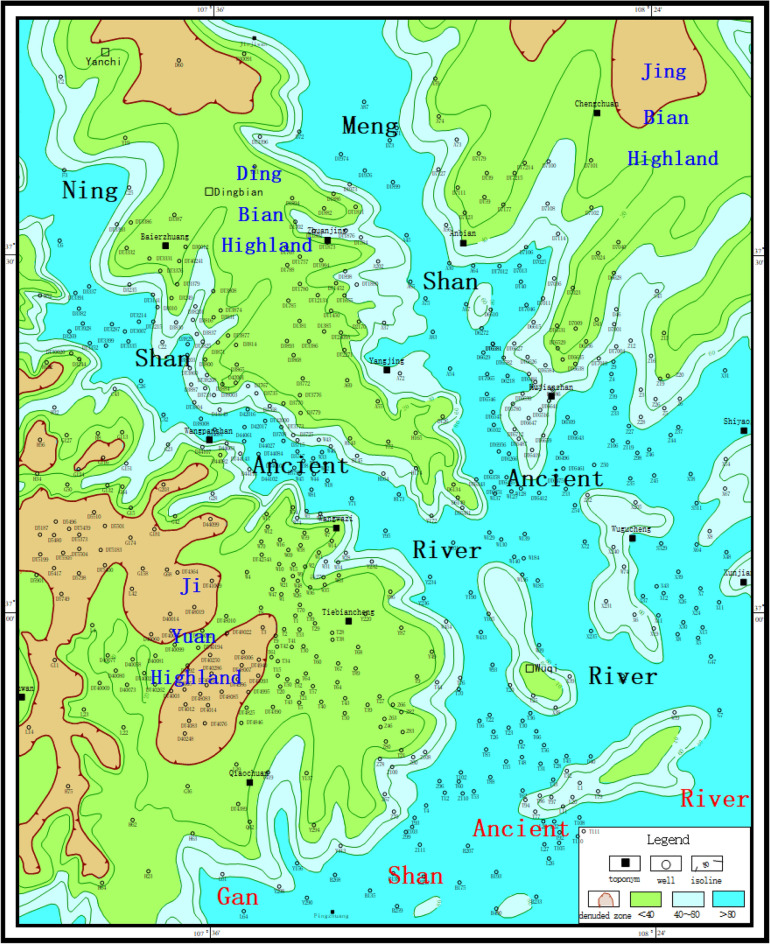
Pre-Jurassic paleogeomorphological map of Wuqi–Dingbian area (Coreldraw, 18.0, https://www.coreldrawchina.com/).

Based on the comprehensive analysis of the horizontal distribution characteristics of sandstone thickness and the pre-Jurassic strata in Yan 10 + Fuxian Formation in the study area, the distribution of ancient water system is depicted to restore the pre-Jurassic paleogeomorphology in Wuding area (Fig. 7). It can be seen that the study area mainly develops four types of ancient geomorphology units, such as ancient river, slope, highland and interriver hill.

#### The Ancient Rive

According to the standard of modern river classification and the scale of ancient rivers in the basin, the ancient rivers in the basin are divided into: the first-level ancient river is the Ganshan Ancient River, and the second-level ancient river is the Ningshan and Mengshan Ancient River. In addition, there are a number of branches with different scales^11^. The Yan 10 + Fuxian Formation in the valley is more than 100 m thick, with the thickest part exceeding 260 m. Typical river filling structure and internal bedding are developed in the valley. The first-order ancient valley of Ganshan and Shaanxi is distributed in the middle of the study area in an almost east–west direction, with a width of 15–40 km and a height difference of 180–260 m from the highland. From west to east, the outcrop strata are the Upper Triassic Chang 6, Chang 4 + 5, Chang 3 and Chang 2 formations, which reflect that the flow direction is from west to east. Ningshan second-order ancient valley spread in the study area in a NW to SE direction, with a width of 10–15 km and a height difference of 140–200 m with an outcrop layer Chang 2. The second-order ancient valley of Meng-shan is nearly north–south, intersecting with the first-order ancient valley of Ganshan from north to south. The valley is 8–12 km wide, with a height difference of 100–160 m from the plateau, and the outcrop layer is Chang 2.

#### Interriver hills

Interfluvial hummocks are residual hummocks formed by erosion and erosion of river valleys, with irregular shapes and higher topography than ancient river channels^12^. The interriver hills in the study area are mainly located in Wuqi area. The Fuxian Formation is relatively thin, with a thickness of 20–30 m in general. The total thickness of the Fuxian Formation and Yan 10 Formation is also relatively thin, with most of them ranging from 60 to 100 m and some areas lower than 70 m, and the height difference between them and the river valley is about 50–120 m.

#### Slope

Slopes are units with a certain slope and relatively high topography, surrounded by ancient river channels^13^. There are 11 slopes in the study area, including the south slope of Jiyuan, the north slope of Jiyuan and the west slope of Jingbian. The slope zone is relatively wide and slow, with a gradient of about 5.7–6.3 m per kilometer. Due to the erosion and cutting of the second-order ancient valley, the slope front is dismembered and broken, its shape is finger-like, and it is commonly called slope nozzle. In these pitts, the sandstone thickness of Yan 10 + Fuxian Formation is 0–110 m, and the sand layer is thinned towards the highlands. It is the main sedimentary site of the beach facies sand body in Yan 10 sedimentary period, and it is the accumulation area of palaeo-geomorphic reservoir.

#### Highland

Highland refers to a terrain unit with higher terrain, and Jiyuan and Dingbian Highlands are mainly developed in the study area^14^. The Fuxian Stage and Yan 10 sedimentary stage suffered from weathering and denudation for a long time, resulting in the absence of sediment or the development of thin floodplain subfacies with a thickness of only 10–20 m. The Jiyuan Highland was cut into seven small elevations isolated from each other by the branch valley, and the outcropping layer was Chang 1. The saddle between these small elevations received the fluvial mud deposits in the late Yan 10 sedimentary period.

Under the control of this paleogeomorphology, the geological characteristics of the pre-Jurassic area are as follows: (1) the outcrop strata in the deep valley are old, up to Chang 6, Chang 4 + 5 and Chang 3, while the stratigraphic strata on the ancient slopes and highlands are new, with Chang 2 and Chang 1; (2) The exposed horizon is closely related to the downcut intensity and river flow direction. In the west (upper reaches), the downcut intensity is high and the emerging horizon is old (Chang 6), while in the east (middle and lower reaches), the downcut intensity is weak and the emerging horizon is new (Chang 2). (3) The new and old changes of emerging horizon reflect the overall uplift of the basin during the Indosinian movement at the end of Triassic. The western and southern emerging horizon has a large old and rising amplitude, while the eastern and northern emerging horizon has a small new and rising amplitude.

### Evolution of paleolandscapes

**Figure 8:**
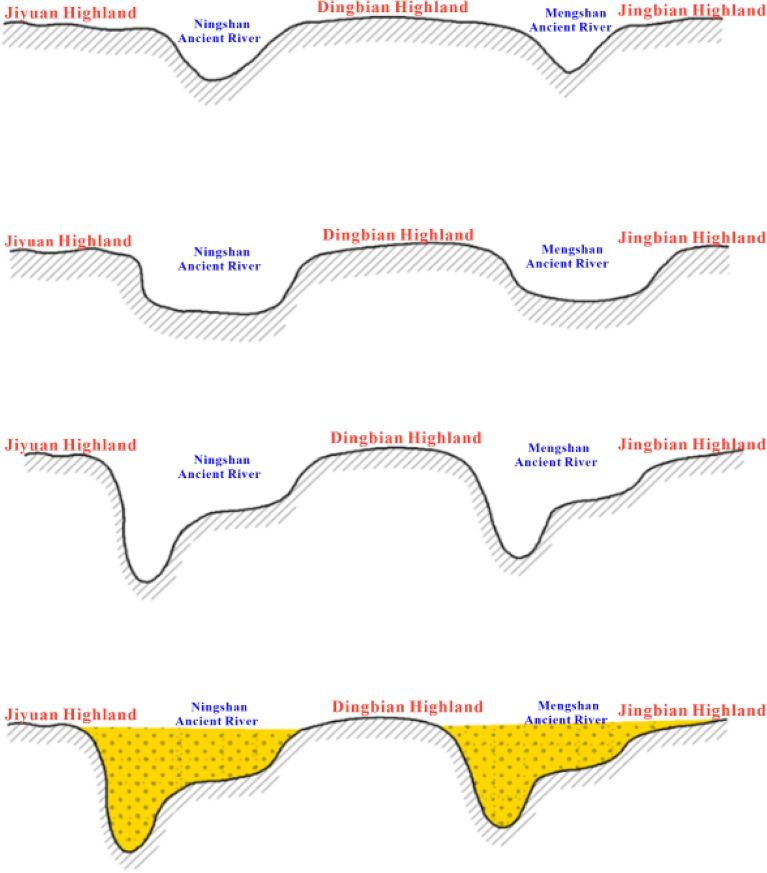
Formation and evolution model of pre-Jurassic paleogeomorphology in Wuqi–Dingbian area.

The paleogeomorphic evolution of the pre-Jurassic period can be generally divided into four stages (Fig. 8).

The first stage: the Late Indochina tectonic activities in the first stage led to the overall uplift of Yanchang Formation, and the erosion channels in Ningshan and Mengshan gradually formed and expanded. The river was dominated by vertical downward erosion, and the valley section was V-shaped. Jiyuan and Dingbian highlands began to take shape.

The second stage: with the uplift erosion gradually tending to the stable equilibrium, the river erosion gradually developed from vertical to lateral erosion, and the valley morphology gradually changed to U-shaped and channel-shaped. The landform of the ancient river and the highlands on both sides was further strengthened, the elevation difference was further enlarged, and the pattern of ancient landform was basically formed.

The third stage: The second stage of tectonic activity led to the differential overall uplift of the underlying strata, which was relatively high in the southwest and northeast of the basin, relatively low in the study area, and the overall structure was V-shaped. The river is close to upland areas.

The fourth stage: shortly after the river erosion, the basement of the basin changed from uplift to subsidence, the river erosion gradually weakened and stopped, and the basin began to settle and fill, and the early Jurassic Fuxian Formation, Yan 10 and other strata gradually filled the ancient valley, until the late deposition of Yan 9 formation, the paleogeomorphology of the basin was basically filled up.

## Control of paleogeomorphology on Jurassic reservoirs

The Wuqi–Dingbian area of Ordos Basin has a complete range of Jurassic palaeo-geomorphic units, including second-order palaeo-river beds, interfluvial mounds, mounds and slopes, among which the interfluvial mounds have large area, multiple levels of mounds and broad slopes. Rich drilling and logging data on each palaeo-geomorphic unit, so it is the most favorable area to study the distribution and control factors of large-scale valley filling erosion palaeo-river reservoirs.

### Paleogeomorphology controls sedimentary facies

Paleotopography is a major factor controlling the development and distribution of sedimentary facies in the study area. Under the influence of palaeo-geomorphology at the end of late Triassic, in the sedimentary period of Fuxian and Yan 10, the branch palaeo-rivers held by the ancient highlands merged into the second and third palaeo-rivers through gentle slopes, the upper terrain received interchannel or interdistributary bay deposits, and the lower terrain received thick channel sand deposits. Starting from the Fuxian Formation, the influence of paleogeomorphology on sediment gradually weakened from the bottom up until the end of the Yan 9 filling and replenishment period^15^.

From Fuxian Formation to Yan 9 formation in Wuqi–Dingbian area, the sediments of gravel braided river, sandy braided river, low curvature meander river and high curvature meander river developed successively. Gravel braided river sediments are mainly gravel channel sediments, and sandy braided river sediments can see gravel bottom scour surface in some areas, but most of them are sandy sediments. Compared with gravel braided river sediments, river terraces are not developed, the downward erosion is weakened, and the river channels are wider and shallower.

For the sand body structure, the thick layer sand body of the ancient channel develops and is continuous longitudinally, and the curve shape is box-shaped, while the thickness of the sand body at the slope position is relatively thinned or a complete multi-stage superposition of the channel binary structure deposit is formed. The curve shape is spindle or drawer. The sand body is not developed in the high position, and the curve shape is often finger-like.

### Paleomorphology controls storage and cover conditions

During the Triassic period, the Ordos Lake Basin occurred and developed on the base of a large platform block with vast waters, wide and flat basement, and uniform depositional conditions. Chang 7 of Yanchang Formation deposited extremely thick deep lacustrine dark oil shale, which entered the peak of hydrocarbon generation in the Late Jurassic and early Cretaceous. Therefore, favorable oil-generating depression provided the oil source basis for the formation of good oil and gas reservoirs in the study area^16–19^.

The basin uplift in the late Indosinian movement led to the denudation of the top strata of the Triassic and the formation of the paleotopography with great fluctuation. The Jurassic strata were deposited directly on these paleotopography and formed unconformity with them. Until the top of Yan 9 was completed, the study area basically returned to the west dip monoclinal structure. On the denudation surface of Yanchang Formation, the thickness of deposits from Fuxian to Yan9 varies. The highlands receive less sediment because of the high location of the ancient landform, and the ancient rivers and slopes receive more sediment because of the low location of the ancient landform. The compressibility of Yanchang Formation is much smaller than that of Yan’an Formation, so the greater the thickness of Jurassic deposition, the greater the compressibility. Therefore, under the influence of differential compaction, the nose structure is easy to form in the slope position.

1. The differential compaction structure is the direction of long-term migration of Jurassic reservoirs and the place of oil and gas accumulation. The Yan 10 reservoir of Jurassic is a channel sand deposit, which is restricted by time and space during deposition, and has poor lamination and rapid lateral change of channel, providing good sedimentary conditions for the formation of lithologic traps. At the same time, the difference in thickness between the unundulated paleo-geomorphic morphology of the pre-Jurassic and the deposition of Yan 10 formation formed the palaeo-geomorphic foundation of the compacted structure, and the nose bulge structure formed by the tectonic stress in the later period provided a favorable direction and good trap for oil and gas^20^.
2. Through comprehensive lithology analysis of Yan 9 member, combined with sedimentary facies and sand body distribution plane characteristics of Yan 9 stage. It is found that a large area of swamping occurred in the study area at the late Yan 9 stage, and a set of gray black mudstone, lacustrine mudstone, carbonaceous mudstone, coal seam and diagenetic dense zone developed, which became a good sealing condition for the trap.
3. According to the reservoir distribution map of Yan 10, Yan 9 and Yan 8 formations, the superposition of micro-amplitude structures at the top of each layer and the analysis of reservoir anatomy, the reservoir accumulation points of Jurassic Yan10-Yan8 formations in Wuqi–Dingbian area are mainly distributed in the high points of each dome structure. It can be seen that the migration points of Jurassic oil and gas are nose uplift structure and differential compaction structure. The direction of oil and gas migration points to the tectonic high point.

Due to the relatively small size of the Yan 10-Yan 8 oil formation sand body in this area, it has a certain influence on the small anticline structure formed by differential compaction in this area. The nasal uplift structure formed in the regional background is superimposed with the low-amplitude anticline structure formed by the different sand body, which plays an important role in controlling the reservoir size. The magnitude of structural trap and the degree of dome development have a certain control effect on the scale and quantity of reservoir formation.

### Palaeo-geomorphic control migration and accumulation condit-ions

The Jurassic Ancient River is an important guarantee to communicate the lower oil source and ensure the upward migration of oil and gas. The top of Yanchang Formation was subjected to long-term leaching weathering and denudation, which formed an obvious erosional terrain. Coupled with further erosion by river channels in the early Jurassic period, the bottom of Yan’an Formation even directly contacted the Chang 3 formation of Yanchang Formation. The Chang 2 formation of Ningshaan-Guhe and Mengshaan-Guhe have emerged, which is undoubtedly a favorable place for the release of excess pressure of Yanchang Formation. The deep valley releases excess pressure of the source reservoir and thus becomes the main channel for oil and gas migration^21,22^. Oil and gas can migrate both vertically and laterally along the channel sand body through the ancient river valley, and accumulate in places with good trap conditions. After the source rock of Chang 7 Yanchang Formation in the study area matured, abnormal pressure was generated inside the source rock to drive oil and gas to migrate upward. The vertical distance of oil and gas migration from Yanchang Formation to Yan’an Formation was greatly shortened by the downward-cut paleo-valley deposited in the paleogeomorphic background, and the high porosity and high permeability sand body deposited in the paleo-valley reduced the migration resistance. Therefore, oil and gas migrated from Yanchang Formation to Yan’an Formation through the high permeability superimposed sand body and the Triassic/Jurassic unconformity in the valley with a high probability, and accumulated in the effective reservoir of Yan’an Formation. The paleogeomorphology communicates the effective matching between Yanchang Formation oil source and Yan’an Formation reservoir, which greatly improves the effectiveness of source-transport-reservoir elements in Yan’an Formation.

### Paleogeomorphology controls reservoir distribution

The oil and gas distribution around the Jurassic palaeoriver landform, the third or fourth grade palaeoriver developed on the slope of the valley, the depth of the palaeoriver valley gradually became shallow and the thickness of the sand body gradually thinned as it extended to the high point of the slope. In the process of migrating upward along the third-grade and fourth-grade ancient river valley, the source of the river channel was encountered, and the connectivity of the sand body became poor, and the sand body settled there and gathered into the reservoir. The source of the secondary channel is often lower than the lower stump of the slope zone, which is also an important place for oil and gas accumulation.

1. Hill mouth landform is a favorable place for lithologic reservoir. Hill mouth is located at the confluence of two ancient rivers^23^, and most of the place is the edge beach facies zone, with high mineral maturity and good physical properties of the sediments. The hill mouth at the confluence of the second and third ancient rivers is located under the water surface due to its low topography, and it is difficult to form reservoir although the channel sand body is developed. The hill mouth at the junction of the third and fourth ancient rivers is located on the slope, the terrain ratio is higher than the water surface, and the small anticline formed by the different compaction of the channel sand body and lithology is developed, and it is easy to form a reservoir.
2. The slope zone is the most important oil storage zone of Jurassic system. The unconformity interface between the river channel and Yanchang Formation on the ancient highland is the main channel for oil and gas migration, and the ancient highland is the most widely distributed part of the Jurassic Yan 9 oil formation and above^24^. The tertiary channel sand body on the slope is still developed, with good connectivity, abundant bottom water and developed unconformity interface. Therefore, driven by the bottom water, the oil and gas in the secondary palaeohanoi migrate to the upper part along the slope and palaeochannel. When reaching the source of the tertiary channel, the oil and gas will enter the overlying Yan 9-Yan 8 oil formation sand body, and accumulate in Yan 9, Yan 8 and even Yan 7 oil formation.

In general, the Jurassic reservoirs show the distribution characteristics of "more slope, less highland, more bottom and less upper part of the ancient river".

### The bottom water driving characteristics are obvious

The Yan 10 and Yan 9 oil formations in the palaeo-valley of the Jura system are like a huge sponge body, with the flow system discharged by post-sedimentary compaction and the infiltration system of atmospheric precipitation and surface water. The former generally migrates from the high pressure area in the valley to the low pressure area on both sides of the valley slope and highland direction. The latter migrated from the high potential energy zone in the upper part of the structure to the low potential energy zone in the lower part, and the alternating activity of the two is one of the important conditions for reservoir enrichment in the Wuqi–Dingbian area. The most active part of bottom water activity is the valley area with large sediment thickness in early Yan 10 and Yan 9, while the bottom water system is relatively stagnant in the erosion plateau and slope area at the edge of the plateau, which is conducive to reservoir preservation. Most of the present Jurassic reservoirs are located on the upper side of the river hills and slopes near the highlands, and the driving, lifting and sealing of oil by the bottom water system is an important factor in the formation of Jurassic reservoirs. Due to the influence of bottom water activity, the oil layer is on the top of the sand body, even if there is no structural control, the lenticular sand body is still a favorable part of the reservoir production.

## Study of reservoir-forming models of palaeo-geomorphic reservoirs

### Slope type

**Figure 9:**
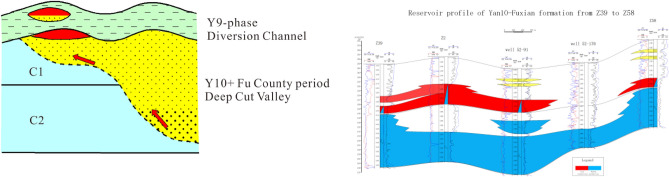
Model diagram of slope reservoir type.

The slope is located in the high part between the valley and the highlands, and the slope zone is the highest slope zone in the pre-Jurassic paleogeomorphic unit. When oil and gas migrated from the river valley along the unconformity or the Jurassic superimposed sand body to the higher part, the slope was the first place where oil and gas arrived. Therefore, when the overlying sedimentary body of Yan'an Formation has a good matching relationship with the slope, it is easy to form a lithologic structural trap, that is, a slope reservoir formation model. The characteristics of this reservoir-forming model are that the longitudinal superposition relationship of sand bodies is not obvious, and the lateral change from old to new sand bodies is rapid. The reservoir-forming is mainly concentrated at the mantle structure of Fuxian and Yan 10, and the reservoir is located on the slope of paleo-geomorphology (Fig. 9).

### Interriver hill type

**Figure 10:**
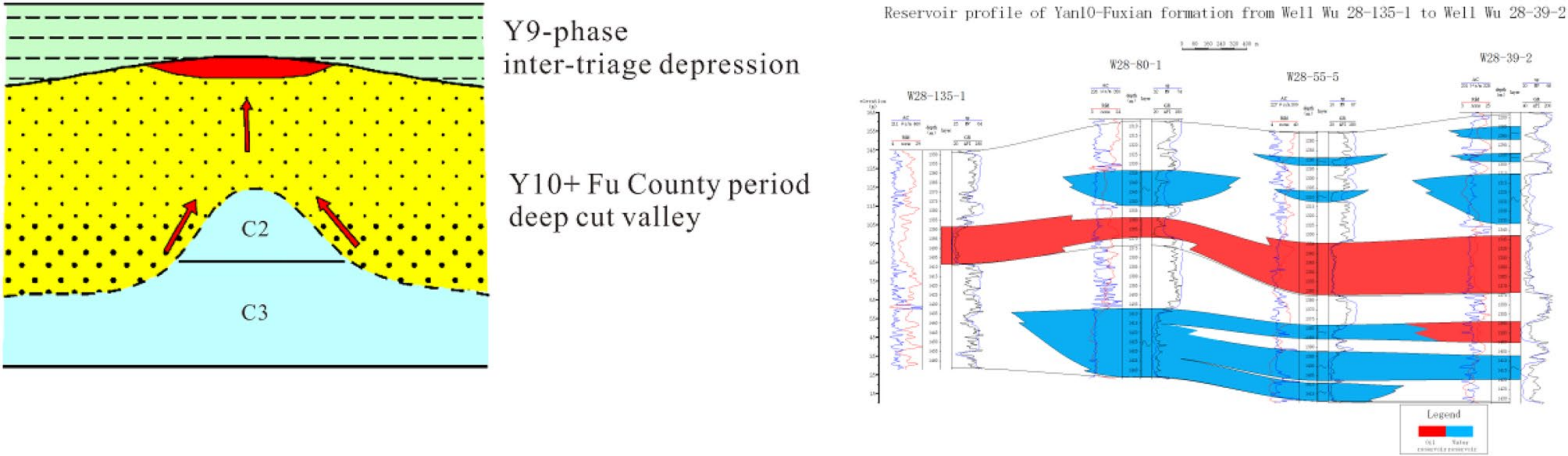
Reservoir type model diagram of interriver hill.

An interfluvial mound is a convex structure at the bottom of the valley. Oil and gas migrated along the upper part of the bulge and migrated through the super thick sandstone of Fuxian and Yan 10 to form the reservoir in the upper part, which became the reservoir formation model of Hejian mound. Yan 10 layers were deposited above it, but the thickness was thin or the local Yan 10 layer was missing. Due to the compaction and the reconstruction of regional tilt in the later period, nose structures were formed on the top of the dome, and a group of lithologic reservoir groups were formed under the control of nose structures. The main type of reservoir was updip lithologic pinch-out reservoir and the second was lens reservoir. The characteristics of this reservoir formation model are that the longitudinal superposition of sand bodies is obvious, the lateral change from old to new sand bodies is little, and the development of overlying sand bodies under producing Wells is relatively thin, showing the characteristics of interfluvial hills. Oil and gas migrated upward from the unconformity surface and hyperpermeability sand body along the interriver hills, and accumulated in the nose uplift of Fuxian and Yan10 (Fig. 10).

### Ancient River type

**Figure 11:**
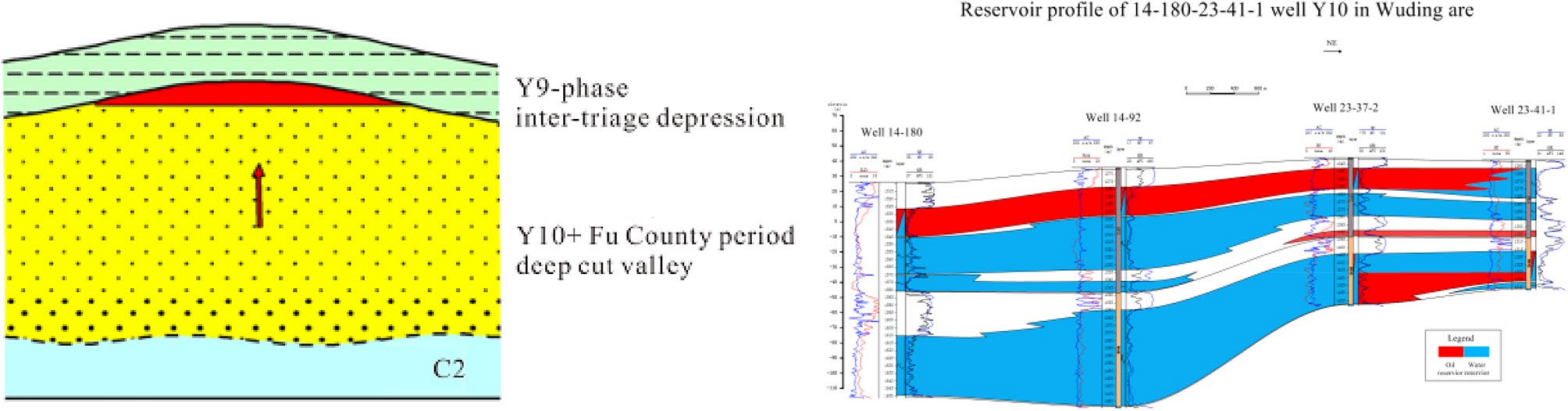
Model diagram of Palaeofluvial reservoir type.

Oil and gas migrated from deep river valleys through superimposed sand bodies. The local nose uplift developed on the ancient river valleys is an effective structural trap, the highly porous and highly permeable channel sand body is a high-quality reservoir for oil and gas accumulation, and the mudstone deposits developed on the floodplain above it are an effective cap layer. When all the above-mentioned reservoir forming factors are present and right-click matched, an effective reservoir can be formed, that is, an ancient river reservoir (Fig. 11).

### Highland type

**Figure 12:**
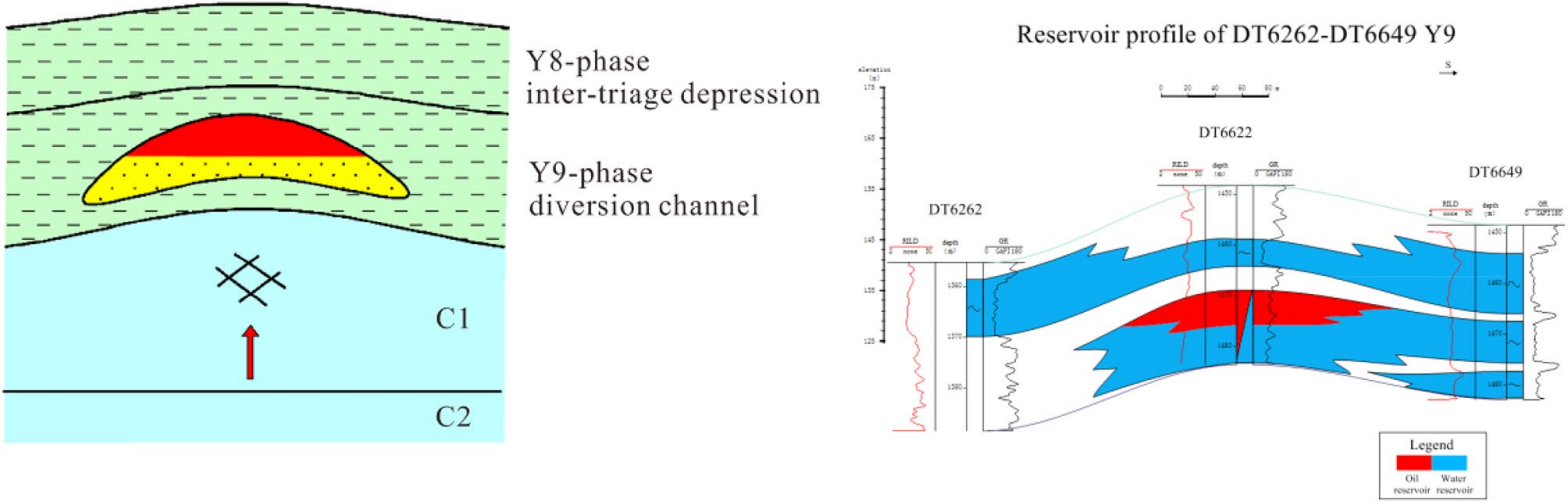
Model diagram of highland reservoir type.

The ancient plateau is the most important reservoir distribution area of Yan 9 member in the study area. When oil and gas migrated to higher locations, the slope could not accumulate to form a reservoir due to the migration force (mainly buoyancy) and other geological factors (reservoir and cap combination, structure), or after a certain amount of oil and gas was trapped in the slope, the plateau was the end of oil and gas migration, and oil and gas could accumulate to form a reservoir in the favorable part of the plateau. At this time, due to sufficient migration force, the formed reservoirs were mostly in a beaded distribution. At the same time, some oil and gas in Yanchang Formation can directly migrate vertically into the trap inside Yan 9 plateau through the dominant unconformity (the effective match between water inflow sand body and semi-weathered sandstone) to form a reservoir (Fig. 12). On the broad and gentle ancient highlands, there are weak tectonic uplifts, mostly nose-shaped structures, on which some lithologic reservoir combinations may be formed. At present, most of the oil reservoirs discovered are single lithologic reservoirs with small area, and scattered distribution.

## Conclusion

1. Through comprehensive restoration of late Triassic paleogeomorphology by multiple methods and fine and three-dimensional characterization of pre-Jurassic paleogeomorphology, it can be seen that there are four types of paleogeomorphology units in the Wuqi–Dingbian area of Ordos Basin in the pre-Jurassic period, including ancient rivers, slopes, uplands and interriver hills, and their paleogeomorphology evolution is generally divided into four stages.
2. The favorable oil-generating depression provides abundant oil resources for the Jurassic reservoir. The relationship between Jurassic palaeogeomorphology and oil content shows that palaeogeomorphology controls sedimentary facies, hydrocarbon generation, accumulation and migration, which indirectly affects the distribution of the oil reservoir. The compacted structure and lithology barrier provide trap conditions for palaeo-geomorphic oil and gas reservoirs, the swamp coal measures and mudstone in the top of Yan9 play a sealing role in oil accumulation, and the deep valley in the pre-Jurassic is the main channel for oil and gas migration.
3. The systematic study of paleo-geomorphic reservoir types and the guidance of reservoir formation models have further clarified the exploration direction of this area. In recent years, the discovered geological reserves of this type of oil have increased from 35 million tons before the study to nearly 100 million tons at present, with the reserves increasing by nearly three times. The discovery of a highland oil reservoir is a new development. As exploration deepens, the potential for this prospect will expand.

## Data Availability

The datasets used and/or analysed during the current study available from the corresponding author on reasonable request.
